# Efficacy of milbemycin oxime/afoxolaner chewable tablets (NEXGARD SPECTRA^®^) against *Capillaria aerophila* and *Capillaria boehmi* in naturally infected dogs

**DOI:** 10.1186/s13071-021-04648-y

**Published:** 2021-03-06

**Authors:** Angela Di Cesare, Simone Morelli, Giulia Morganti, Giulia Simonato, Fabrizia Veronesi, Mariasole Colombo, Michele Berlanda, Wilfried Lebon, Matilde Gallo, Frederic Beugnet, Donato Traversa

**Affiliations:** 1 Faculty of Veterinary Medicine, University of Teramo, 64100 Teramo, Italy; 2 Department of Veterinary Medicine, University of Perugia, 06126 Perugia, Italy; 3 Department of Animal Medicine, Production and Health, University of Padua, 35020 Padua, Legnaro Italy; 4 Boehringer-Ingelheim Animal Health, Lyon, France

**Keywords:** *Capillaria aerophila*, *Capillaria boehmi*, Dog, Milbemycin oxime, NEXGARD SPECTRA^®^

## Abstract

**Background:**

*Capillaria aerophila* and *Capillaria boehmi* parasitize the respiratory system of wild and domestic carnivores. *Capillaria aerophila* inhabits the trachea and bronchi of dogs and cats, while *C. boehmi* affects the nasal cavities and sinuses of dogs. In dogs the infection may be subclinical or characterized by varying respiratory distress.

**Methods:**

The present study evaluated the efficacy of an oral formulation containing milbemycin oxime and afoxolaner (NEXGARD SPECTRA^®^) in dogs naturally infected with *C. aerophila* and/or *C. boehmi* from three enzootic areas of Italy. Dogs were enrolled pending fecal examination and molecular confirmation of respiratory capillarioses. Dogs were allocated in two groups: Group 1 (G1, 25 dogs), treated with a negative control product with no anthelmintic activity (afoxolaner, NEXGARD^®^), and Group 2 (G2, 26 dogs), treated with NEXGARD SPECTRA^®^. At the day of treatment administration (Day 0), all dogs were clinically examined. Dogs were again subjected to clinical and fecal examinations at Days 28 (± 4) and 56 (± 2). The primary criterion for treatment efficacy was the reduction of fecal *Capillaria* egg counts in G2 compared with G1. The regression of/recovery from baseline clinical signs was considered as a further efficacy criterion.

**Results:**

Percentage reduction of fecal *Capillaria* egg counts in the NEXGARD SPECTRA^®^ group compared to the control group was > 97% on Day 28 and 100% on Day 56, respectively (*p* < 0.05 for both time points). Twelve of the 13 dogs in the NEXGARD SPECTRA^®^ group with respiratory signs prior to treatment were free of clinical signs at the end of the study. Conversely, the six control group dogs with respiratory signs prior to treatment remained symptomatic.

**Conclusions:**

Results of the present study showed that NEXGARD SPECTRA® was safe and highly efficacious in the reduction of *C. aerophila* and *C. boehmi* eggs after one treatment with a complete reduction of the egg output after the second administration associated with a recovery from respiratory signs. 
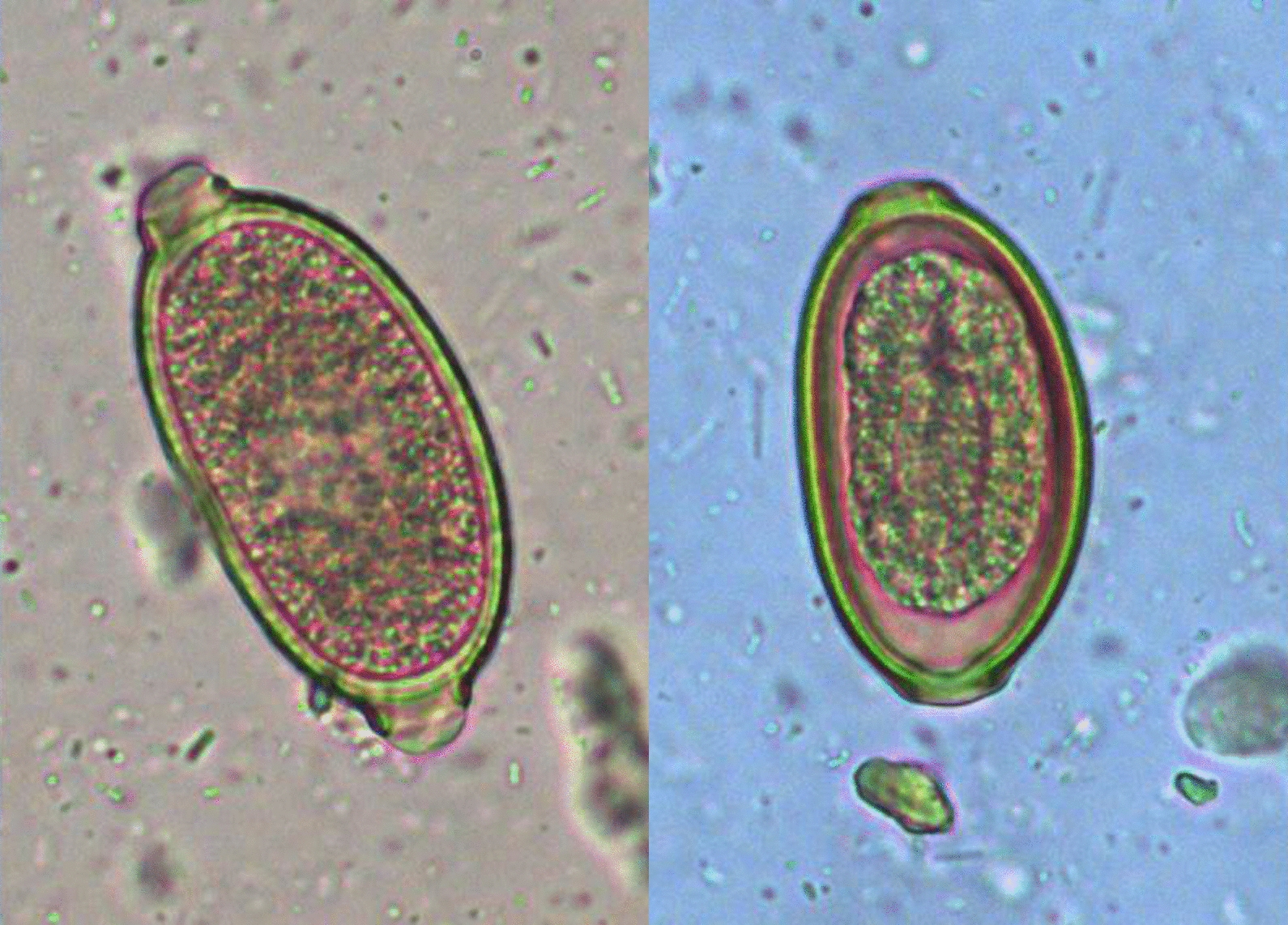

## Background

The trichuroid nematodes *Capillaria aerophila* and *Capillaria boehmi* are responsible for respiratory infections in various carnivores. Adults of *C. aerophila* live underneath the epithelium of trachea and bronchi of wild carnivores, dogs, cats, mustelids and occasionally humans, while those of *C. boehmi* inhabit the nasal turbinates and the frontal and paranasal sinuses of wild canids and dogs [1–3]. Adult females lay eggs that are swallowed and shed *via* the feces in the environment. The definitive host acquires the infection by ingesting larvated eggs and/or earthworms acting as paratenic or facultative intermediate hosts [4].

Infections by *C. aerophila* and *C. boehmi* are increasingly reported in domestic carnivores, probably as a result of the access to wild environments and the arrival of wildlife to urban and peri-urban areas [3, 5–12]. Depending upon parasite burden and microbial co-infections, dogs infected by *C. aerophila* may present a subclinical picture or pulmonary capillariosis characterized by chronic bronchitis, bronchopneumonia and respiratory failure. Most common clinical signs are chronic productive or dry cough, sneezing, wheezing, ocular/nasal discharge and bronchovesicular sounds [5, 13]. Analogously, *C. boehmi* infections may be subclinical or result in chronic conditions, especially in the case of heavy parasite burden. Clinical signs include sneezing (reverse or not), serous to purulent nasal discharge, epistaxis and hypo- or anosmia [1, 14–16]. Cerebral damage due to intracranial migrations of parasite elements has been described [17].

Despite a rise in records in various countries of Europe [7, 18–22], only limited therapeutic options are available. NEXGARD SPECTRA® (Boehringer Ingelheim Vetmedica GmbH) is an oral combination of the isoxazoline afoxolaner and the macrocyclic lactone milbemycin oxime (MO). This formulation is licensed for the treatment and prevention of a broad spectrum of canine ecto- and endoparasites. Afoxolaner is efficacious against major fleas, ticks and mites in dogs, while MO provides efficacy against intestinal and extra-intestinal nematodes. MO has been reported to be efficacious against canine capillarioses [18, 20, 23, 24]. The present study aimed to assess the efficacy of MO contained in NEXGARD SPECTRA^®^ for the treatment of naturally acquired *C. aerophila* and/or *C. boehmi* infections in dogs under field conditions.

## Methods

### Study design

This study was a multi-centric negative-controlled field trial, using a blinded model with a randomized block based on order of presentation/enrollment of dogs. The general study design followed the International Cooperation on Harmonisation of Technical Requirements for Registration of Veterinary Medicinal Products—VICH GL7 “Efficacy of Anthelmintics: General Requirements” [25] and VICH GL19 “Efficacy of Anthelmintics: Specific Recommendations for Canines” [26]. The study was approved by the Italian Health Ministry (authorization no. 330202290—23/04/2019-DGSAF) and was conducted in three sites located in Central (Site A and B, Umbria and Abruzzo regions) and Northern Italy (Veneto region, Site C).

### Pre-inclusion screening

Dogs were screened for *C. aerophila* and/or *C. boehmi* eggs using a conventional floatation technique [27] within 23 and 7 days prior to treatment. For each positive dog, two fecal samples collected from two consecutive defecations between 6 to 2 days before treatment were subjected to a McMaster method using a 1.350 specific gravity zinc sulfate solution (sensitivity 50 eggs per gram of feces, EPG) [27] to obtain the baseline (pre-treatment) fecal *Capillaria* egg count. In addition, *Capillaria* eggs were identified to species based on size and morphological characters, i.e. asymmetry of bipolar plugs, presence/absence of space between the embryo and the wall and the characteristics of the outer shell [1, 28]. Samples positive for *C. aerophila* and/or *C. boehmi* eggs at the conventional floatation technique were also analyzed using PCR to confirm microscopic identification with two diagnostic nested PCRs [29, 30].

### Inclusion/exclusion criteria and study enrollment

With the molecular confirmation and the baseline EPG values, each dog was evaluated for additional criteria prior to enrollment in the study.

Dogs that satisfied the following inclusion criteria were enrolled: dogs ≥ 8 weeks old, ≥ 2.0 kg; dogs clinically healthy except from signs of respiratory parasitic infection; dogs tested negative for *Dirofilaria immitis* antigen (i.e. *FAST*est® HW Antigen, Megacor).

Exclusion criteria were: debilitated dogs and/or those suffering from disease or injuries; aggressive dogs; pregnant female or female dogs intended for breeding; dogs that had received drugs containing any product with anthelmintic activity within 60 days before the study start.

### Clinical examination, allocation and treatments

On Day 0, prior to treatment administration, all dogs were clinically examined, with focus on upper (i.e. sneezing, reverse sneezing, scent impairment, catarrhal blood-stained or muco-purulent nasal discharge) and lower (i.e. chronic cough, dyspnea, general respiratory distress, bronchovesicular sounds) respiratory tract signs. Thereafter, each animal was assigned to one of the two groups with a 1:1 ratio, negative control (G1) or NEXGARD SPECTRA^®^ (G2) based on a randomization list provided by the sponsor. G1 dogs received NEXGARD^®^ (providing no anthelmintic activity) while G2 dogs received NEXGARD SPECTRA^®^ at Days 0 and 28 (± 4), based on individual body weight and per label instructions.

### Post-treatment evaluation and efficacy criteria

All dogs were clinically and copromicroscopically re-evaluated at Days 28 (± 4) and 56 (± 2). Fecal egg counts were established by using a modified McMaster technique (sensitivity 50 EPG) [27]. To increase the sensitivity, the copromicroscopic examinations were performed on two fecal samples collected from two consecutive defecations. Two subsamples were taken from each of two consecutive defecations for examination so that per occasion results of four subsamples per dog were averaged for the individual fecal egg count (individual *Capillaria* EPG) to be considered. Dogs were considered negative if no eggs were counted in all four subsamples.

The primary efficacy criterion was the post-treatment egg count reduction per time point of G2 compared with G1.

Individual *Capillaria* EPG counts were transformed to the natural logarithm of (count + 1) for calculation of geometric means for each treatment group. Percent efficacy was calculated using Abbott’s formula: 100 *  [(*C* − *T*)/*C*], where *C* is the geometric mean of the control group G1 and T is the geometric mean of the NEXGARD SPECTRA^®^ group G2.

The counts of the G2 group were compared to the counts of the G1 group at each time point (untransformed data) using a non-parametric Wilcoxon rank sum test. All testing was two-sided at the significance level *α* = 0.05.

The post-treatment regression or recovery of clinical signs from baseline was considered a further efficacy criterion.

Dogs copromicroscopically positive for *Capillaria* spp. eggs on day 56 (± 2) received an appropriate rescue treatment at the discretion of the responsible veterinarian for each site.

## Results

Overall, 189 privately owned dogs were screened. Among them, 138 dogs tested negative for *Capillaria* eggs and 51 dogs testing positive for *Capillaria* eggs were enrolled in the study, i.e. 20, 20 and 11 at Sites A, B and C, respectively. Twenty-five and 26 dogs were allocated to groups G1 and G2, respectively. All dogs completed the study according to the protocol, with no adverse event, and all were included in the efficacy calculations. The copromicroscopic/molecular analysis demonstrated *C. aerophila* eggs in 35 dogs (68.6%), *C. boehmi* eggs in 14 dogs (27.5%) and both *C. aerophila* and *C. boehmi* eggs in 2 dogs (3.9%). Details on species detected and treatments administered at each study site are shown in Table 1. Throughout the study, all dogs in G1 tested positive for fecal *Capillaria* eggs to at least at one copromicroscopic examination at each time point.

**Table 1:** Number of dogs infected with *Capillaria aerophila* (*C. a.*), *Capillaria boehmi* (*C. b.*) or both in Sites A–C

Site	Group	Number of positive dogs on Day -6/-2			
		*C. a.*	*C. b.*	*C. a.* and *C. b.*	Total
A	1	6	4	0	10
	2	6	4	0	10
B	1	10	0	0	10
	2	10	0	0	10
C	1	2	2	1	5
	2	1	4	1	6
Total	1	18	6	1	25
	2	17	8	1	26

Group 1: dogs treated with the negative control product NEXGARD^®^

Group 2: dogs treated with NEXGARD SPECTRA^®^

Among the dogs in G2, 15 (57.7%) dogs were copromicroscopically negative on Day 28 (± 4) while 11 dogs shed *Capillaria* eggs (7 dogs *C. aerophila* and 4 dogs *C. boehmi*). All G2 dogs were negative for *Capillaria* eggs on Day 56 (± 2).

Compared to the control group G1, NEXGARD SPECTRA^®^-treated dogs had significantly lower *Capillaria* egg counts at each post-treatment evaluation. Percentage reductions were 97.5% and 100% at Days 28 (± 4) and 56 (± 2), respectively (Table 2). Thus, two NEXGARD SPECTRA^®^ treatments administered 1 month apart showed completely stopped shedding of eggs of both *C. aerophila* and *C. boehmi*.

**Table 2:** Geometric mean of EPG for *Capillaria* spp. at each time point and percent of reduction at each time point

	Day 6/-2	Day 28	Day 56
Group 1	120.39 [0–600]	94.04 [0–300]	127.38 [0–1400]
Group 2	164.11 [0–1350]	2.36 [0–100]	0.00 [0–0]
Efficacy (*P*-value)	NA (NS)	97.49% (1.65 × 10^−9^)	100.00% (2.12 × 10^−11^)

Group 1: dogs treated with the negative control product NEXGARD^®^

Group 2: dogs treated with NEXGARD SPECTRA^®^

*NS* Not statistically significant. [Minimum–maximum EPG values]

Pre-treatment clinical examination revealed clinical signs potentially related with respiratory *Capillaria* spp. infection in 6/25 (24%) and 13/26 (50%) dogs from G1 and G2, respectively. Chronic dry cough was the most common clinical sign observed in dogs shedding *C. aerophila* eggs [4/25 (16%) and 7/26 (26.9%) dogs from G1 and G2, respectively]. Nasal discharge was the most common clinical sign in observed dogs shedding *C. boehmi* eggs [3/26 (11.5%) from G2]. On the Day 56 (± 2) examination, 12/13 (92.3%) dogs of G2 had no more respiratory signs, while the number of G1 dogs presenting respiratory signs increased from 6 (Days 0 and 28 ± 4) to 8 (Days 56 ± 2) (Table 3).

**Table 3:** Number (*n*/tot) and percentage (%) of dogs infected with *Capillaria aerophila* and/or *Capillaria boehmi* showing one or more clinical signs related to upper/lower respiratory tract on Day 0, Day 28 and Day 56

Clinical sign	Group	Day 0	Day 28	Day 56
Sneezing	1	1/25	1/25	1/25
	2	3/26	0/26	0/26
Reverse sneezing	1	1/25	1/25	1/25
	2	0/26	1/26	0/26
Nasal discharge	1	0/25	0/25	0/25
	2	4/26	3/26	0/26
Bronchovesicular sounds	1	0/25	0/25	2/25
	2	3/26	0/26	0/26
Chronic dry cough	1	4/25	4/25	4/25
	2	7/26	2/26	1/26
Dry cough	1	0/25	0/25	1/26
	2	0/26	0/26	0/26
Number of dogs with at least one clinical sign	1	6/25 (24%)	6/25 (24%)	8/25 (32%)
	2	13/26 (50.0%)	6/26 (23.1%)	1/26 (3.8%)

Group 1: dogs treated with the negative control product NEXGARD^®^

Group 2: dogs treated with NEXGARD SPECTRA^®^

## Discussion

The results of the present study indicate that the combination product containing MO and afoxolaner (NEXGARD SPECTRA^®^) had a beneficial effect for treating pulmonary and nasal capillarioses in infected dogs with two consecutive monthly treatments. This study confirms previous reports on the efficacy of MO against *Capillaria* infections in dogs under field conditions [3, 18, 20, 23, 24]. A > 99% efficacy of NEXGARD SPECTRA^®^ in reduction of egg shedding in dogs has been previously published, although the eggs were not identified to species [18]. A recent study that investigated the combined monthly use of NEXGARD SPECTRA^®^ and fipronil/permethrin (FRONTLINE TRI-ACT^®^, Boehringer Ingelheim Vetmedica GmbH) against endo- and ectoparasites of dogs indicated 100% efficacy against *C. aerophila* in 8 dogs 14 days after treatment [20]. The therapeutic efficacy of MO against *C. boehmi* was also reported in single clinical cases [23, 24]. Off-label therapeutic options, e.g. fenbendazole administered in different dosages and treatment protocols, have been proposed but always assessed in few cases [31–33]. To date, no products are licensed in the EU for treating canine pulmonary capillariosis, while a topical combination formulation containing moxidectin has the indication for treating *C. boehmi* infection in dogs [16, 34]. The therapeutic efficacy of moxidectin and eprinomectin against feline pulmonary capillariosis has been demonstrated in GCP studies [35–37], and a topical formulation containing moxidectin claims to treating *C. aerophila* infection in cats [35–37].

The post-treatment recovery for respiratory signs of 12/13 symptomatic dogs treated with NEXGARD SPECTRA^®^ in the present study supports the high efficacy demonstrated based on fecal egg count reduction. Further studies based on a combination of copromicroscopic, clinical, genetic and endoscopic tools could be of interest to ultimately demonstrate the adulticidal activity of MO. The persisting dry cough in one NEXGARD SPECTRA^®^-treated dog may be related to severe chronic lesions, which may require more time for a complete recovery or other underlying conditions.

Results of the present study are of relevance to canine veterinary medicine as the availability of a product with efficacy against both pulmonary capillariosis and nasal capillariosis would be greatly appreciated in clinical settings. Both parasites are often present in the same areas, and mixed infections are relatively frequent in dogs living in enzootic regions ([8, 29, 30, 38, 39], present results). Relevance for practitioners is also associated with the fact that differential diagnosis of *C. aerophila* and *C. boehmi* infections based on egg morphology requires a specific training and can be challenging [33, 38, 40]. This is of importance in those cases when misidentification between the two parasites occurs, as an efficacious product would be administered in any case.

Foxes are frequently infected by *C. aerophila* and *C. boehmi* in Europe, e.g. Serbia [8, 41], Bosnia and Herzegovina [10], Austria [11], Switzerland [33]**,** Germany [42] and Italy [9, 43, 44]. Accordingly, hunting dogs and dogs with outdoor access living in peri-urban areas where foxes are present are under particular risk of infection with *Capillaria* spp. [45, 46]. Indeed, more than half of the herein enrolled dogs were kept for hunting (i.e. 31 dogs), and all with the exception of two dogs living in urban areas with free access to the house garden lived in the countryside, always outdoors or with free/regular outdoor access. These categories of dogs are particularly exposed to several parasitic infections and vector-borne diseases [46–51]. In these animals, NEXGARD SPECTRA^®^ may be particularly useful against a wide range of ectoparasites, along with the control of many nematodes of major importance.

According to recent scientific knowledge and the 3Rs concept [52], the present data provide further evidence that in vivo examinations, including follow-up of the clinical picture and quantitative assessment of parasite excretion under natural conditions may be suitable approaches to the assessment of the efficacy of anthelmintics in dogs.

## Conclusions

Results of the present study showed that NEXGARD SPECTRA^®^ was highly efficacious in the reduction of *C. aerophila* and *C. boehmi* eggs after one treatment (97.5%) with a complete reduction of the egg output after the second administration and that the product was safe and efficacious for the treatment of canine pulmonary and nasal capillarioses.

## Data Availability

All relevant data generated or analyzed during this study are included in this published article.

## Abbreviations

EPG: Egg per gram of feces
G1: Group 1, dogs treated only with a negative control product containing afoxolaner (NEXGARD^®^) with no anthelmintic activity
G2: Group 2, dogs treated with an oral formulation containing milbemycin oxime and afoxolaner (NEXGARD SPECTRA^®^)
MO: Milbemycin oxime
PCR: Polymerase chain reaction
